# The choice of negative control antisense oligonucleotides dramatically impacts downstream analysis depending on the cellular background

**DOI:** 10.1186/s12863-021-00992-1

**Published:** 2021-09-14

**Authors:** Luca Ducoli, Saumya Agrawal, Chung-Chau Hon, Jordan A. Ramilowski, Eliane Sibler, Michihira Tagami, Masayoshi Itoh, Naoto Kondo, Imad Abugessaisa, Akira Hasegawa, Takeya Kasukawa, Harukazu Suzuki, Piero Carninci, Jay W. Shin, Michiel J. L. de Hoon, Michael Detmar

**Affiliations:** 1 Institute of Pharmaceutical Sciences, Swiss Federal Institute of Technology (ETH) Zurich, Vladimir-Prelog-Weg 3, 8093 Zurich, Switzerland; 2 Molecular Life Sciences PhD Program, Swiss Federal Institute of Technology and University of Zurich, Zurich, Switzerland; 3 RIKEN Center for Integrative Medical Sciences, Yokohama, Kanagawa 230-0045 Japan; 4 RIKEN Center for Life Science Technologies, Yokohama, Kanagawa 230-0045 Japan; 5 RIKEN Preventive Medicine and Diagnosis Innovation Program, RIKEN Center for Life Science Technologies, Yokohama, Kanagawa 230-0045 Japan; 6 Human Technopole, Via Cristina Belgioioso 171, 20157 Milan, Italy

**Keywords:** Antisense oligonucleotide, ASO, CAGE-Seq, Cap analysis of gene expression, Long noncoding RNA, lncRNA

## Abstract

**Background:**

The lymphatic and the blood vasculature are closely related systems that collaborate to ensure the organism’s physiological function. Despite their common developmental origin, they present distinct functional fates in adulthood that rely on robust lineage-specific regulatory programs. The recent technological boost in sequencing approaches unveiled long noncoding RNAs (lncRNAs) as prominent regulatory players of various gene expression levels in a cell-type-specific manner.

**Results:**

To investigate the potential roles of lncRNAs in vascular biology, we performed antisense oligonucleotide (ASO) knockdowns of lncRNA candidates specifically expressed either in human lymphatic or blood vascular endothelial cells (LECs or BECs) followed by Cap Analysis of Gene Expression (CAGE-Seq). Here, we describe the quality control steps adopted in our analysis pipeline before determining the knockdown effects of three ASOs per lncRNA target on the LEC or BEC transcriptomes. In this regard, we especially observed that the choice of negative control ASOs can dramatically impact the conclusions drawn from the analysis depending on the cellular background.

**Conclusion:**

In conclusion, the comparison of negative control ASO effects on the targeted cell type transcriptomes highlights the essential need to select a proper control set of multiple negative control ASO based on the investigated cell types.

**Supplementary Information:**

The online version contains supplementary material available at 10.1186/s12863-021-00992-1.

## Background

Tight control of gene expression at several levels is a crucial prerequisite for maintaining gene plasticity, responsiveness to environmental changes, and ensuring proper development. The vasculature, composed of blood and lymphatic vessels, undergoes an intricate series of regulatory mechanisms to safeguard the physiological functioning of the organism. Increased activation or impaired function of these vascular networks can contribute to the development of severe pathological conditions such as cancer, chronic inflammatory diseases, diseases leading to blindness, metabolic syndrome, atherosclerosis, and neurodegeneration [1, 2]. Many efforts have been invested in understanding the role of signaling, transcriptional, and post-transcriptional as well as post-translational regulators in the regulation and maintenance of identity and function of lymphatic and blood vascular endothelial cells (LECs and BECs) [1, 2]. However, very few studies were undertaken to elucidate the role of long noncoding RNAs (lncRNAs) in LEC and BEC biology.

During the last decades, the FANTOM (Functional Annotation of the Mammalian Genome) consortium made striking contributions to the discovery and characterization of the lncRNAs by demonstrating, through Cap Analysis of Gene Expression (CAGE-Seq), that the human genome is constitutively transcribed, producing various sense and antisense transcripts [3]. Subsequent efforts revealed that the lncRNA family constitutes approximately 72% of the transcribed genome [4]. In general, lncRNAs are categorized according to their genomic location and orientation relative to protein-coding genes [5]. lncRNAs are either classified as intergenic (lincRNA), intronic, antisense noncoding transcripts based on the protein-coding genes in their genomic neighborhood, and promoter- or enhancer-derived based on epigenetic markers at their promoters [6–8]. In addition to that, the increasing evidence that lncRNAs are involved in various aspects of gene expression regulation emphasizes the relevance of lncRNA classification based on their functions [9, 10]. In the nucleus, lncRNA transcripts can act either locally (*in cis*) or on different chromosomes (*in trans*), primarily as a scaffold for various functional protein complexes involved in transcriptional regulation, chromatin remodeling, or RNA processing [11–14]. Moreover, some lncRNA genes do not function through their transcribed RNA molecules but rather through their simple act of transcription [11–14]. This can influence the transcription of neighboring genes by altering epigenetic states as well as the recruitment of the transcriptional machinery. On the other hand, in the cytoplasm, lncRNAs can also function as a scaffold for protein complexes regulating mRNA stability, translation, and decay [11–14]. This vast functional repertoire of lncRNAs has led to the novel idea of RNA as a central molecule in the regulation of gene functions. Specific expression patterns of lncRNA subsets have also been associated with cell state coordination, cell differentiation, development, and disease progression [15, 16]. Moreover, mutation and/or overexpression of lncRNAs have been implicated in a multitude of human diseases, proposing lncRNA signatures as possible diagnostic factors of malignant conditions [17].

To explore the functional role of lncRNAs in LECs or BECs, we performed antisense oligonucleotide-mediated knockdown (ASOKD) of four lncRNA candidates, previously identified as LEC- or BEC-specific lncRNAs, followed by CAGE-Seq [18]. Here, we present the early quality control steps adopted in the analysis pipeline prior to determining the transcriptional changes after lncRNA target KD in either LECs or BECs. Through this quality check, we assessed the negative impact on LEC proliferation of one commercially available negative control ASO and, therefore, excluded it from our analysis. In addition, to our best knowledge, our dataset represents the first source of information on the transcriptional impacts of lncRNA KDs in human LECs or BECs and, therefore, will be a valuable resource for the vascular community for further studies aiming to characterize the functionality of lncRNAs in LECs and BECs.

## Results

### ASO-mediated knockdown transcriptomic profiling of lineage-specific lncRNAs

**Fig. 1:**
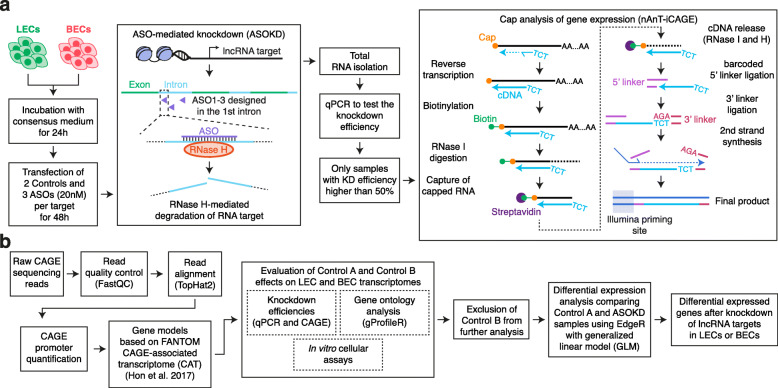
Overview of the experimental procedure. **(a)** Schematic representation of the experimental workflow. LECs and BECs were subjected to ASO-mediated knockdown (ASOKD) followed by Cap Analysis of Gene Expression (CAGE-Seq). Only samples with a knockdown efficiency higher than 50% in both replicates were subjected to CAGE-Seq. **(b)** Bioinformatic pipeline highlighting the quality control steps prior to the transcriptome profiling after lncRNA candidate knockdowns

Figure 1 shows the experimental design and the bioinformatic control-step workflow before characterizing the transcriptional impacts of 2 LEC and 2 BEC lncRNA target knockdowns. LECs and BECs were first transfected in duplicates with eight ASOs independently (negative control A and B and three ASO per lncRNA target; Additional file 1). Only samples with KD efficiency higher than 50% in both replicates for at least one primer pair were subjected to CAGE-Seq (Fig. 1a). Finally, after mapping and CAGE promoter quantification, the impacts of negative control ASOs on LECs and BECs were evaluated by performing Differential Expression (DE) and Gene Ontology (GO) analysis and in vitro cellular assays (Fig. 1b).

Our dataset comprised 32 CAGE-Seq libraries, as described in Additional file 2. After removing low-quality sequencing reads, each library contains, on average, a total of 15 million reads. In the majority, 93% of reads were mapped to the genome, confirming the high quality of the analyzed samples (Additional file 2).

### Negative control ASOs display similar knockdown efficiencies of lncRNA targets

**Fig. 2:**
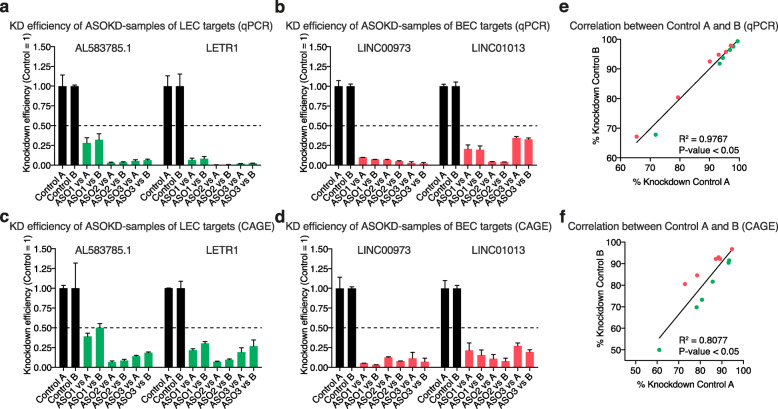
Quality control of knockdown efficiencies after lncRNA knockdown. **(a-d)** Comparison of knockdown efficiencies after knockdown of 2 LEC and 2 BEC lncRNAs using either negative control A or B, as determined by qPCR (a, b) or CAGE-Seq (c, d). Data are represented as mean values + SD (*n* = 2). **(e, f)** Correlation of knockdown efficiencies between negative control A and B, as determined by qPCR (e) and CAGE-Seq (f). *P*-values were calculated using linear regression

To evaluate the effects of negative control ASOs (A or B) on LECs and BECs, we first compared the KD efficiencies for each ASO targeting the lncRNA candidates using both negative control ASOs individually as reference. Both qPCR and CAGE-Seq techniques confirmed that all samples had a KD efficiency higher than 50% regardless of the negative control ASO used (Fig. 2a-d). However, we also observed that referencing to either negative control A or B led to slight differences in the degree of the KD efficiencies in our CAGE-Seq data compared to qPCR results (Fig. 2a-d). In LEC samples, negative control A led to a slightly higher KD efficiency than negative control B (Fig. 2c). Vice versa, BEC samples displayed a higher KD trend after comparing to negative control B (Fig. 2d). This finding was further supported by correlation analysis between negative control A and B KD efficiencies, where a lower but still significant correlation was observed in CAGE-Seq data in comparison to qPCR results (Fig. 2e, f). Despite these minor differences, we concluded that both negative control ASOs were suitable for determining the ASO-mediated knockdown efficacy in both cell types.

### Negative control B causes deregulation of genes associated with LEC proliferation

**Fig. 3:**
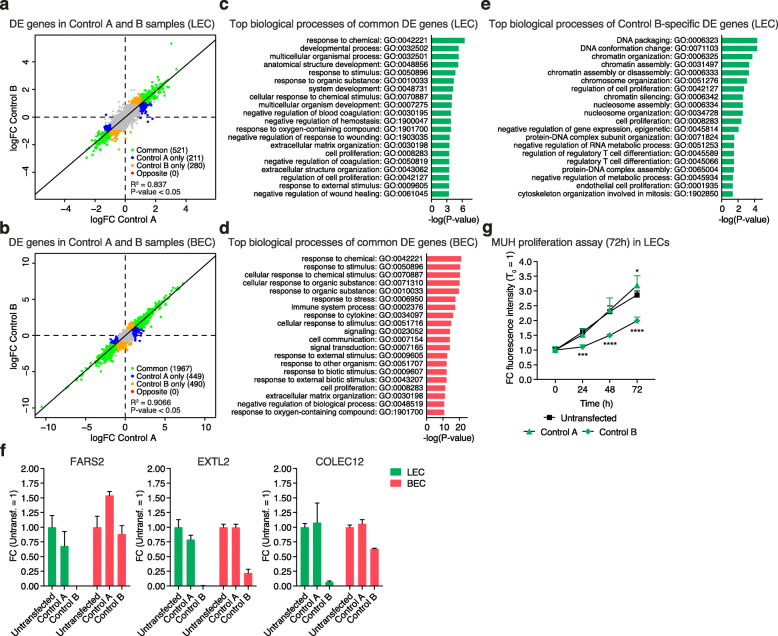
Quality control of the transcriptional impact of negative controls on LEC or BEC transcriptome. **(a, b)** Correlation of log2FC between differentially expressed (DE) genes in negative control A and B. Green dots: DE genes in common between negative control A and B; blue and orange dots: specific to either negative control A or B; red dots: opposite pattern (red). *P-*values were calculated using linear regression. **(c-e)** Top significantly (*P-*value < 0.05) enriched GO terms for biological processes of commonly DE genes between negative control A and B in LECs and BECs (c, d), and specific DE genes for negative control B (e), using g:ProfileR [19] (relative depth 1–5). GO terms were ordered according to -log(*P-*value) values. **(f)** Expression levels of FARS2, EXTL2, and COLEC12 in LECs and BECs after transfection with negative control A and B. Bars represent fold change (FC) values against untransfected cells. **(g)** Quantification of the 4-methylumbelliferyl heptanoate (MUH) proliferation assay over 72 h in neonatal LECs derived from the same donor after negative control A or B transfection. Dots represent FC of the fluorescence intensity against T_0_. In f and g, data are displayed as mean values + SD (*n =* 2 in f and *n* = 5 in g). In g, *P-*value: * < 0.05, *** < 0.001, **** < 0.0001, using two-way ANOVA with Dunnet’s multiple comparisons test against untransfected control. The in vitro assay was performed in neonatal LECs derived from the same donor

Next, we investigated whether the effects of ASO transfection on the general transcriptome of LECs or BECs were consistent between the two negative control ASOs by comparing them to the untransfected reference CAGE-Seq samples (Additional file 2, refer to the original study [18] for further details). For the comparison, we considered only genes displaying a | log2FC| > 1 and an FDR corrected *P*-value < 0.05. The results showed that perturbation using negative control A and B caused the deregulation of 744 (up: 430; down: 314) and 813 (up: 454; down: 359) genes in LECs and 2487 (up: 1371; down: 1116) and 2487 (up: 1383; down: 1104) in BECs (Additional file 3). The FC values of the DE genes were largely overlapping between both negative control ASOs (Fig. 3a, b and Additional file 4), which is likely to be attributable to the lipofectamine treatment as previously observed in human dermal fibroblasts [20]. Further, GO enrichment analysis of the DE genes common between negative control A and B showed, in both cell types, an enrichment for biological processes associated mainly with responding to external factors (Fig. 3c, d). Hence, these changes are likely to be effects of lipofectamine treatment. However, based on the current experimental settings, we cannot completely exclude that some of these changes are also due to impacts intrinsically connected to both negative control ASOs.

The results also showed that each negative control ASO caused the deregulation of a specific subset of genes (Additional file 4). Additional GO enrichment analysis revealed that negative control B-specific DE genes in LECs were enriched for various biological processes (Fig. 3e), primarily related to chromatin organization and endothelial cell proliferation. However, no GO terms for biological processes were observed to be significantly enriched in negative control A-specific DE genes in LECs or negative control A/B-specific DE genes in BECs.

### Negative control B inhibits LEC proliferation in vitro

Given this enrichment on cell proliferation-related terms, we then analyzed empirically whether negative control B was affecting the ability of LECs to proliferate. First, we confirmed, through qPCR, the higher reduction in LECs than BECs of three top downregulated negative control B-specific genes (FARS2, EXTL2, and COLEC12) previously involved in the positive regulation of cell proliferation and physiology [21–25] (Fig. 3f). Interestingly, COLEC12 has been previously reported as a novel lymphatic endothelial cell marker, further supporting the cell type-specific effect of negative control B in LECs [26, 27]. Second, 4-methylumbelliferyl heptanoate (MUH) proliferation assay showed that negative control B transfection significantly inhibited the proliferation of LECs (Fig. 3g). Based on these results, we therefore decided to exclude negative control B CAGE-Seq libraries from further analyses and only use negative control A to investigate the lncRNA candidate knockdown effects on the transcriptome of either LECs or BECs (Fig. 1b, refer to the original study [18] for further details).

## Discussion

This study complements our previous findings, where we analyzed the functionality of human lncRNAs in vascular biology by performing ASO-mediated knockdown of 2 LEC- and 2 BEC-specific lncRNAs followed by CAGE-Seq [18]. Here, we presented the early control steps in which we carefully characterized the transcriptional impact on LECs and BECs of two commercially available negative control ASOs. In particular, we revealed that specifically in LECs, the negative control B exerted off-target effects that included genes associated with LEC biology. Furthermore, although referencing to either negative control A or B showed comparable lncRNA candidate knockdown levels, we described via in silico and in vitro analyses that the lipofectamine-based delivery of negative control B significantly inhibited LEC proliferation by deregulating several proliferation-related genes. Overall, we present an efficient pipeline to detect confounding factors associated with negative control ASO transfections that can significantly influence the interpretation of the results in different cellular backgrounds.

Since studies involving ASOs in characterizing lncRNA function are increasing [28, 29], future investigators must be aware of the potential challenges encountered when comparing their ASO knockdown data to negative control ASOs. Although very valuable commentaries have been published in the past [30–33], there are still very high discrepancies on how to properly use ASO in studying a target of interest.

Based on our results, we therefore recommend selecting multiple negative control ASOs to have a minimum of two controls that are not impacting the general transcriptome and cellular function of the target cell types. We also advise choosing at least three ASOs targeting the lncRNA candidates that show similar knockdown efficiencies. As mentioned above, in this study, we used two commercially available negative control ASOs that ideally should not bind any sequence present in the tested cells. Besides that, we also suggest including alternative negative control ASOs such as mismatched sequences that abrogate the binding to the target sequence. Moreover, the fact that one negative control caused dramatic changes in one of the tested cell types adds an extra layer of complexity that needs to be carefully evaluated. We therefore strongly encourage the research community to closely inspect the off-target effects of the chosen set of negative controls on their respective experimental cellular backgrounds. Along with the necessary repetition of the experimental procedure, these guidelines will help design coherent lncRNA knockdown studies leading to a solid interpretation of lncRNA knockdown effects.

In addition to these guidelines, we strongly suggest the inclusion of previously reported instructions in the experimental design [30, 31]. First, we recommend including not only libraries from untreated cells but also lipofectamine-only treated cells to evaluate the potential effects of the transfection reagent on the cell of interest [31]. Second, analysis of gap ablated ASO and ASO backbone modification data can provide further useful information on the off-target effects of the negative control ASOs and to differentiate between off-target cleavage and steric hindrance [34–36]. Third, a rigorous evaluation of dose-response and time-course experiments will help determine the best experimental conditions and provide direct comparisons between experiments [30]. Finally, measuring cellular uptake through microscopy or testing the target cleavage by biochemical techniques (such as 5′/3′-RACE) is an additional layer of control experiments that can support the proper localization and gene knockdown of the target of interest [30, 37].

Future studies should also consider alternative delivery methods of the ASOs. For instance, previous studies showed the possibility of delivering ASOs by gymnosis [38, 39]. In one study, naked ASOs were efficiently delivered to the target cell without any delivery vehicle by carefully controlling the plating conditions and the duration of the experiment. However, we agree that performing a large-scale knockdown experiment that satisfies all the presented requirements can be extremely costly and dependent on the availability of lab resources.

As a next step, the transcriptomic profiling results need to be supported by thorough biochemical and mechanistic studies [29]. For instance, observed molecular phenotypes must be corroborated by in vitro cellular assays using ASO and orthogonal techniques, such as short interference RNAs (siRNAs) and/or CRISPR interference (CRISPRi). In our recent studies, we efficiently connected the molecular phenotypes associated with the lncRNA target knockdown, as predicted by the CAGE-Seq data analysis, with essential cellular functions and provided detailed evidence on their molecular mode of actions combining RNA-DNA, RNA-protein, and RNA-chromatin interaction studies [18, 20].

## Conclusion

In conclusion, the present study analyzed the effects of negative control ASOs on the transcriptome of LECs and BECs. We provide evidence that a careful evaluation of the differential expression pattern of negative control ASO transfections is an essential step before performing subsequent downstream analyses. Furthermore, despite the congruency in knockdown efficiency estimations, we observed that one of the selected commercially available negative control ASO caused unwanted side effects in LECs, affecting their viability. Thus, we pinpoint the essential need to accurately examine multiple negative control ASOs in order to select a proper control set with no to minimal effects on the transcriptome of the targeted cell types. Taken together, our study, in conjunction with previously published guidelines and case studies, represents practical advice for precisely studying lncRNA function using ASOs [30–33].

## Methods

### ASO knockdown in LECs and BECs and sample preparation for CAGE-Seq

Primary human dermal lymphatic and blood vascular endothelial cells (LECs and BECs) were collected from neonatal foreskin. LECs and BECs were isolated as previously described [40] and expanded in complete endothelial basal medium (EBM (Lonza), 20% FBS, 100 U/mL penicillin and 100 μg/mL streptomycin (Pen-Strep, Gibco), 2 mM L-glutamine (Gibco), 10 μg/mL hydrocortisone (Sigma)) on 10 cm dishes (TPP) pre-coated with 50 μg/mL purecol type I bovine collagen solution (Advanced BioMatrix) in DPBS (Gibco) at 37 °C in a 5% CO_2_ incubator. LECs were additionally cultured in the presence of 25 μg/mL cAMP (Sigma); BECs in the presence of endothelial cell growth supplement ECGS/H (PromoCell). At passage 7, 7 × 10^5^ LECs and 6 × 10^5^ BECs were seeded into 10 cm dishes and cultured overnight. The next day, medium was exchanged with 8 mL consensus medium (EBM, 20% FBS, 100 U/mL penicillin and 100 μg/mL streptomycin (Pen-Strep), 2 mM L-glutamine), and both cell types were cultured for an additional 24 h. LECs and BECs were then transfected with a mixture of 20 nM ASO (1–3 ASOs per target or negative control A or B transfected individually, GeneDesign) and 16 μL Lipofectamine RNAiMAX (Thermo Fisher Scientific) in 1.6 mL Opti-MEM (Gibco) following the manufacturer’s instructions and incubated for 48 h (Fig. 1a). The list of ASO sequences used in the study is reported in Additional file 1. LECs and BECs were harvested, and total RNA was isolated using the RNeasy mini kit (Qiagen). DNA digestion was performed using the RNase-free DNase set (Qiagen). RNA was then quantified and checked for quality using NanoDrop ND-1000 (Witec AG). KD efficiency for each ASO was checked by qPCR. According to the manufacturer’s instructions, equal amounts of total RNA were reverse transcribed using the High Capacity cDNA Reverse Transcription kit (Applied Biosystems). 10 ng cDNA per reaction were then subjected to qPCR using PowerUp SYBR Green Master mix (Applied Biosystems) on a QuantStudio 7 Flex Real-Time PCR system (Applied Biosystems). For qPCR analysis, cycle threshold (Ct) values were normalized to the housekeeping gene GAPDH. Relative expression was calculated according to the comparative Ct method. Samples with at least 50% KD efficiency in both replicates were subjected to CAGE-Seq (Fig. 1a). Primers are listed in Additional file 5. KD efficiency was also confirmed by comparing CAGE-Seq data for knockdown and corresponding control samples (Fig. 2).

### Cap analysis of gene expression (CAGE-Seq)

CAGE-Seq was performed according to the nAnT-iCAGE protocol, as previously described [41] (Fig. 1a). Purified total RNA (4 μg) was first subjected to reverse transcription using anchored random primers and Superscript III reverse transcriptase (Thermo Fisher Scientific) for 30s at 25 °C and 1 h at 50 °C. After purification with the Agencourt RNAClean XP kit (Beckman Coulter), cDNA biotinylation was performed as follows. In a first step, cDNA was diol oxidized with 45.4 mM NaOAc (pH 4.5) and 11.3 mM NaIO_4_ for 45 min on ice in the dark. Once the reaction was stopped by adding 1.33% glycerol and 233 mM Tris-HCL (pH 8.5), cDNA was purified as above and then subjected to biotinylation by incubating with 83.3 mM NaOAc (pH 6.0) 0.83 mM Biotin hydrazide for 2 h at 23 °C. RNase I treatment was performed on purified cDNA samples using 5 units (U) RNase ONE ribonuclease (Promega) for 30 min at 37 °C. In the meantime, tRNA-coated magnetic beads were prepared by adding 3.75 μg of tRNA (Sigma) to 150 μg Dynabeads M-270 streptavidin beads (Thermo Fisher Scientific) and incubated for 30 min on ice. tRNA-coated magnetic beads were then washed twice with wash buffer A (4.5 M NaCl, 50 mM EDTA (pH 8.0), 0.1% Tween20), and resuspended in wash buffer A containing 3.75 μg tRNA. The capped RNA capture was performed by incubating RNase I-treated cDNA with t-RNA-coated magnetic beads for 30 min at 37 °C. Next, the beads were washed with several buffers: once with wash buffer A, once with 37 °C preheated wash buffer B (10 mM Tris-HCl (pH 8.5), 1 mM EDTA (pH 8.0), 0.5 M NaOAc (pH 6.1), 0.1% Tween20), and once with 37 °C preheated wash buffer C (0.3 M NaCl, 1 mM EDTA (pH 8.0), 0.1% Tween20). To release 5′ cDNA, beads were incubated twice with release buffer (1x RNaseONE buffer (Promega), 0.01% Tween20) for 5 min at 95 °C. Eluted 5′ cDNA was then incubated with 6 U RNase H (Thermo Fisher Scientific) and 20 U RNase ONE ribonuclease for 15 min at 37 °C in order to release the cDNA fragment from the complementary RNA strand. Single-stranded cDNA was then purified with Agencourt AMPure XP kit (Beckman Coulter) and subjected to another RNase I (5 U) treatment for 30 min at 37 °C. After an additional purification step, cDNA concentration was measured using Quant-iT OliGreen ssDNA reagent and kit (Thermo Fisher Scientific), and the ratio of mRNA/rRNA was analyzed by performing qPCR with ACRB-specific primers and 18S ribosomal cDNA primers on a 7900HT real-time system (Applied Biosystems). Once these quality checkpoints were passed, cDNA was first ligated to barcoded 5′ linkers (2 μM) in DNA ligation mighty mix (Takara Biotech) and incubated overnight at 16 °C. Following another purification step, the 3’linker was then analogously ligated to the 5’linker-ligated cDNA overnight at 16 °C. After overnight incubation, cDNA was purified again and subjected to shrimp alkaline phosphatase (1 U, Affymetrix) for 30 min at 37 °C. Then, 2 U USER enzyme (New England Biolabs) were added to the SAP-treated cDNA and further incubated for 30 min at 37 °C followed by 5 min at 95 °C. Ligated cDNA was purified again and subjected to second-strand synthesis by incubating with 1x ThermoPol reaction buffer pack (New England Biolabs), 0.2 mM dNTPs, 1 mM nAnT-iCAGE 2nd primer, 2 U DeepVent (exo-) DNA pol (New England Biolabs) for 5 min at 95 °C, 5 min at 55 °C, and 30 min at 72 °C. After exonuclease I (20 U, New England Biolabs) digestion for 30 min at 37 °C, purified cDNA sample quality was assessed for linker dimers using Agilent Bioanalyzer (Agilent Technologies), and its concentration was measured using Quant-iT PicoGreen dsDNA reagent and kit (Thermo Fisher Scientific). At this point, 3 ng of samples were finally loaded to the cluster generation. Libraries were combined in 8-plex using different barcodes and subjected to 50-base single-end sequencing on a HiSeq 2500 instrument (Illumina).

### Alignment, transcript assembly, and CAGE-Seq promoter quantification of CAGE-Seq data

Figure 1b displays the bioinformatic analysis pipeline. In the first step, raw sequencing reads were subjected to read quality control using standard pipelines [42]. Trimmed reads were mapped to the human genome assembly hg38 using TopHat2 (ver. 2.0.12) [43] applying default settings (Additional file 2). After alignment, the expression for CAGE-Seq promoters was estimated as previously described [20].

### Evaluation of negative controls a and B effects on LEC and BEC transcriptomes

In the first step, KD efficiencies of lncRNA candidates determined by qPCR and CAGE-Seq were compared between negative control A and B in the corresponding cell types by performing a linear regression analysis by fitting linear models in R. For qPCR, KD efficiencies were calculated according to the comparative Ct method. For CAGE-Seq, on the other hand, KD efficiencies were estimated from normalized count per million (CPM) values.

Next, to study the effects of negative control A or B transfection on LECs and BECs, differential expression (DE) analysis was performed by comparing negative control ASO samples individually against CAGE-Seq libraries from untransfected cells, used in the original study [18] to determine lncRNAs specifically expressed in either LECs or BECs (termed reference libraries in Additional file 2). Genes with expression > = 5 CPM in at least two CAGE-Seq libraries (negative control ASOs (A or B) + reference CAGE-Seq libraries) were defined as expressed genes and were tested for DE using EdgeR (ver. 3.12.1) [44, 45]. Genes with | log_2_ fold change (log2FC)| > 1 and FDR corrected *P*-value < 0.05 were defined as differentially expressed genes and used for the downstream analysis (Additional file 3). Common DE genes were selected with | log2FC| > 1 and FDR < 0.05 cutoffs in both negative control ASOs. Negative control A or B-specific DE genes were defined as log2FC > 1 and FDR < 0.05 in negative control A or B and log2FC < 1 in negative control B or A for upregulated genes; and log2FC < − 1 and FDR < 0.05 in negative control A or B and log2FC > − 1 in negative control B or A for downregulated genes (Additional file 4). Finally, GO analysis was performed on DE genes common between negative control A and B or DE genes specific to either negative control A or B, using g:Profiler (ver 0.6.7) [19] with the Ensembl 90, Ensembl Genomes 37 (rev 1741, build date 2017-10-19) database. All the expressed genes in each cell type were used as background. GO terms with *P-*value < 0.05 were used for further analysis.

### qPCR of selected negative control B-specific genes

35,000 LECs per well were seeded into a 12-well plate and cultured overnight. LECs were then transfected with 20 nM of negative control A or B and 1 μL Lipofectamine RNAiMAX previously mixed in 100 μL Opti-MEM according to the manufacturer’s instructions. RNA isolation, cDNA synthesis, and qPCR were performed as described above. Primers are listed in Additional file 5.

### 4-methylumbelliferyl heptanoate (MUH) proliferation assay

7 × 10^5^ LECs at passage 7 were seeded into 10 cm dishes and cultured overnight in a 5% CO_2_ incubator. The next day, LECs were transfected with 20 nM of negative control ASO A or B and 16 μL Lipofectamine RNAiMAX previously mixed in 1.6 mL Opti-MEM according to the manufacturer’s instructions and incubated for 24 h. Transfected LECs were then detached and seeded at a 3000 cells/well density into a collagen-coated 96-well plate (plack plate, Costar). At each time point, LECs were washed with DPBS (Thermo Fisher Scientific), and 100 μL of 0.1 mg/mL MUH (Sigma) in DPBS were added to each well. The plate was incubated for 1 h at 37 °C. Finally, fluorescence intensities were measured using a SpectraMay Gemini EM system (Molecular Devices) and the SoftMax Pro software (ver. 4.7.1). Excitation, emission, and sensitivity were set to 355 nm, 460 nm, and 14, respectively.

## Supplementary Information

**Additional file 1.** List of ASO sequences.

**Additional file 2.** List of CAGE-Seq libraries with corresponding sequencing statistics.

**Additional file 3.** Differential expressed genes of negative control ASOs (A and B) against untransfected reference control in BECs.

**Additional file 4.** Common, NCA only, and NCB only differential expressed genes of negative control ASOs against untransfected reference control in BECs.

**Additional file 5.** List of primers for qPCR.

**Additional file 6.** Codes used to perform differential expression analyses of CAGE-Seq data.

## Data Availability

All raw sequencing data after the knockdown of the 2 LEC and 2 BEC lncRNAs have been deposited to the DDBJ DRA database. The data can be accessed through the project accession number DRA009940 (https://www.ncbi.nlm.nih.gov/sra/?term=DRA009940). The processed data are available at the following link: https://fantom.gsc.riken.jp/6/datafiles/. The codes used to perform the differential expression analysis of either lncRNA candidate knockdown against negative control ASO samples or negative control ASO against reference libraries are available as Additional file 6.

## Abbreviations

LEC: Lymphatic endothelial cell
BEC: Blood vascular endothelial cell
ASO: Antisense oligonucleotide
CAGE: Cap analysis of gene expression
lncRNA: Long noncoding RNAs
FANTOM: Functional annotation of the mammalian genome
DE: Differential expression
GO: Gene ontology
MUH: 4-methylumbelliferyl heptanoate
CPM: Count per million
