# Supplementary material for: The choice of negative control antisense oligonucleotides dramatically impacts downstream analysis depending on the cellular background
Source: BMC Genom Data. 2021 Sep 14;22:33. doi: 10.1186/s12863-021-00992-1 (PMC8439024; doi:10.1186/s12863-021-00992-1)
Supplement: Supplementary file 6 — Additional file 6. Codes used to perform differential expression analyses of CAGE-Seq data. [file 12863_2021_992_MOESM6_ESM.pdf]

The choice of negative control antisense oligonucleotides dramatically impacts downstream analysis depending on the cellular background

Luca Ducoli, Saumya Agrawal, Chung-Chau Hon, Jordan A. Ramilowski, Eliane Sibler, Michihira Tagami, Masayoshi Itoh, Naoto Kondo, Imad Abugessaisa, Akira Hasegawa, Takeya Kasukawa, Harukazu Suzuki, Piero Carninci, Jay W. Shin, Michiel J. L. de Hoon, Michael Detmar

Additional File 6

Codes used to perform differential expression analyses of CAGE-Seq data

1. Differential expression analysis of negative control ASOs and reference untransfected control

```
library("edgeR")
library("statmod")

setwd("../Ducoli and Agrawal et al - LETR1 DataDescriptor Suppl. File 1")

#-------------Read the ASO details----------------------------------------

ref_details <- read.csv("reference_lib_details.txt" , header = TRUE, sep = "\t")
KD_details  <- read.csv("KD_lib_details.txt" , header = TRUE, sep = "\t")
Crtl_details <- KD_details[ KD_details$manual_sample_info.perturb_id %in% c ("NC_A" , "NC_B") , ]

cell_line = c("BEC","LEC")


for (i in 1:length(cell_line))
{

  #-------------Read raw count---------------------------------------------

  raw_count <- read.delim(paste( as.character(cell_line[i]) , "_gene_count.tsv" , sep=""), 
check.names=FALSE, stringsAsFactors=FALSE)

  #-------------Library details ---------------------------------------------


  cell_ref_details <- ref_details [ref_details$set_info.cell_type %in% cell_line[i],]
  scramble_details <-  Crtl_details [ Crtl_details$set_info.cell_type_alias %in% cell_line[i],]

  scramble_name <- c ("NC_A" , "NC_B")

  for (j in 1:length(scramble_name))
  {
    system(paste("mkdir -p ../" , cell_line[i], "/ref_vs_crtl/" , scramble_name[j],sep="" ))

    dir_name=paste("../" , as.character(cell_line[i]) , "/ref_vs_crtl/" , as.character(scramble_name[j]) , "/"
, sep="" )

    #-------Reference lib name---------------------------------------------------
    cell_ref_libs <- cell_ref_details$library_name

    #-------Scramble lib name---------------------------------------------------
    scramble_libs <- scramble_details$library_name
[scramble_details$manual_sample_info.perturb_id %in% as.character(scramble_name[j])]


    #-------Reference raw count --------------------------------------------
    ref_raw_count <- raw_count[as.character(cell_ref_libs)]
```

```
#-------Scramble Raw count ---------------------------------------------------
scramble_raw_count <- raw_count[as.character(scramble_libs)]

#------- Combine Reference +  scramble raw data--------------------------------

count_data <- cbind(ref_raw_count , scramble_raw_count)
count_data_flt <- count_data[rowSums(count_data >1)>3 , ]
gene_flt <- as.data.frame(raw_count[rowSums(count_data >1)>3,1] )


#------- Filter the raw count ------------------------------------------
keep_cpm <- rowSums(cpm(count_data_flt)>=5)>2
count_cpm_flt <- count_data_flt [keep_cpm,]
#------- Gene name for filtered raw counts-------------------------------
gene_cpm_flt <- as.data.frame(gene_flt[keep_cpm,1])
colnames(gene_cpm_flt) <- "ID"


#------- Generate DG list object ---------------------------------------
count_obj <- DGEList(counts = count_cpm_flt , genes = gene_cpm_flt)
count_obj$samples$group <- c("Ref" , "Ref", "Scramble", "Scramble")


#------- Calculate normalized factor ------------------------------------
count_obj <- calcNormFactors(count_obj)

#------- Multi-dimensional scaling plot----------------------------------

pdf( paste (dir_name, as.character(cell_line[i]) , "_" , as.character(scramble_name[j])   ,"_MDS.pdf",
sep=""))
plotMDS(count_obj , col = c(rep("red",2), rep("black",2)) , top = 1000)
invisible(dev.off())

#------- Desigin matrix-------------------------------------------------

ASO <- factor(c("Ref" , "Ref", "Scramble", "Scramble"),levels=c("Ref" , "Scramble"))
design <- model.matrix(~0+ASO)
colnames(design) <- levels(ASO)

write.table (design, file=paste ( dir_name , as.character(cell_line[i]) , "_" ,
as.character(scramble_name[j])  , "_desigin.txt" ,sep="") , sep="\t", col.names = TRUE, quote =
FALSE, row.names = TRUE)

#------Biological coffcient of variation (BCV) plot --------------------

pdf( paste ( dir_name , as.character(cell_line[i]) , "_" , as.character(scramble_name[j])  ,
"_dispersion.pdf" ,sep=""))
count_obj <- estimateDisp(count_obj, design, robust=TRUE)
plotBCV(count_obj)
invisible(dev.off())

#----------Differential expression analysis------------------------------------------------
fit <- glmFit(count_obj, design , robust = TRUE)
glm_obj <- glmLRT(fit,contrast = c(-1,1))
DE_all <- topTags(glm_obj , n=10000000000000)
```

```
   write.table (DE_all$table, file=paste ( dir_name , as.character(cell_line[i]) , "_" ,
as.character(scramble_name[j])  , "_DE_genes.txt" ,sep="") , sep="\t", col.names = TRUE, quote =
FALSE, row.names = FALSE)

   #----------CPM count---------------------------------------------------------------------
   cpm_value_obj <- cpm(count_obj)
   cpm_value_raw <- cpm(count_data)
   write.table (cbind(count_obj$genes , cpm_value_obj), file=paste ( dir_name ,
"DE_normalized_cpm.tsv" ,sep="") , sep="\t", col.names = TRUE, quote = FALSE, row.names =
FALSE)
   write.table (cbind(raw_count[,1], cpm_value_raw), file=paste ( dir_name , "DE_raw_cpm.tsv"
,sep="") , sep="\t", col.names = TRUE, quote = FALSE, row.names = FALSE)

   #---------Summary file---------------------------------------------------------------------
   DGE_summary <- decideTestsDGE(glm_obj, p.value=0.05)
   c1 <- "Sample information"
   c2 <- "~~~~~~~~~~~~~~~~~~~~~~~~~~~~~~~~~~~~~~~~~~~~~~~~~~~~~~~~"
   c3 <- "DE gene summary"

   sink(file = paste ( dir_name , as.character(cell_line[i]) , "_" , as.character(scramble_name[j])  ,
"_DE_summary.txt" ,sep=""))
     cat (c1)
     cat("\n")
     cat (c2)
     cat("\n")
     print (count_obj$samples)
     cat("\n")
     cat (c2)
     cat("\n")
     cat (c3)
     cat("\n")
     cat (c2)
     cat("\n")
     print (summary(DGE_summary))
     cat("\n")
     cat (c2)
   sink(file=NULL)

    DE_genes <- DE_all$table
    DE_genes_FDR_flt <- DE_genes [ DE_genes$FDR <= 0.05 , ]
    DE_genes_FC_flt <- DE_genes_FDR_flt [ abs(DE_genes_FDR_flt$logFC) >= 1 , ]

   }
}
```

2. Differential expression analysis of lncRNA-targeting ASOs vs negative controls

```
library("edgeR")
library("statmod")

setwd("../Ducoli and Agrawal et al - LETR1 DataDescriptor Suppl. File 1")

#-------------Read the ASO details-----------------------------------------

KD_details  <- read.csv("KD_lib_details.txt" , header = TRUE, sep = "\t")
Crtl_details <- KD_details[ KD_details$manual_sample_info.perturb_id %in% c ("NC_A" , "NC_B") , ]
ASO_details <- KD_details[ !KD_details$manual_sample_info.perturb_id %in% c ("NC_A" , "NC_B") ,
]

cell_line = c("BEC","LEC")


for (i in 1:length(cell_line))
{

  #--------------Read raw count-----------------------------------------------
  raw_count <- read.delim(paste(as.character(cell_line[i]) , "_gene_count.tsv" , sep=""),
check.names=FALSE, stringsAsFactors=FALSE)

  #--------------Library details ---------------------------------------------
  cell_crtl_detail <- Crtl_details [ Crtl_details$set_info.cell_type %in% cell_line[i] , ]
  cell_ASO_detail <- ASO_details [ ASO_details$set_info.cell_type %in% cell_line[i] , ]
  gene_name <- unique(as.character(cell_ASO_detail$geneID))

  for (j in 1:length(gene_name))
  {
    scramble_name <- c ("NC_A" , "NC_B")
    for ( k in 1: length(scramble_name))
    {
      system(paste("mkdir -p ../" , as.character(cell_line[i]) , "/DE_analysis/" , gene_name[j], "/",
as.character(scramble_name[k]) , "/" , sep="" ))
      dir_name=paste("../" , as.character(cell_line[i]) , "/DE_analysis/" , gene_name[j], "/",
as.character(scramble_name[k]) , "/" , sep="" )
      ASO_lib_detail <- cell_ASO_detail[cell_ASO_detail$geneID %in% gene_name[j] , ]
      Crtl_lib_detail <- cell_crtl_detail[cell_crtl_detail$manual_sample_info.perturb_id %in%
scramble_name[k] , ]

      #-------Control raw count --------------------------------------------
      crtl_raw_count <- raw_count[as.character(Crtl_lib_detail$library_name)]

      #-------ASO Raw count ------------------------------------------------
      ASO_raw_count <- raw_count[as.character(ASO_lib_detail$library_name)]

      #------- Combine Reference +  scramble raw data-------------------------------
      count_data <- cbind(crtl_raw_count , ASO_raw_count)
      count_data_flt <- count_data[rowSums(count_data >1)>7 , ]
      gene_flt <- as.data.frame(raw_count[rowSums(count_data >1)>7,1] )

      #------- Filter the raw count ------------------------------------------
      keep_cpm <- rowSums(cpm(count_data_flt)>=5)>2
      count_cpm_flt <- count_data_flt [keep_cpm,]

      #------- Gene name for filtered raw counts------------------------------
```

```
	gene_cpm_flt <- as.data.frame(gene_flt[keep_cpm,1])
	colnames(gene_cpm_flt) <- "ID"

	#------- Generate DG list object ---------------------------------------
	count_obj <- DGEList(counts = count_cpm_flt , genes = gene_cpm_flt)
	count_obj$samples$group <- c( as.character( Crtl_lib_detail$manual_sample_info.perturb_id ),
as.character(ASO_lib_detail$manual_sample_info.perturb_id))

	#------- Calculate normalized factor ----------------------------------
	count_obj <- calcNormFactors(count_obj)

	#------- Multi-dimensional scaling plot--------------------------------
	pdf( paste (dir_name, as.character(gene_name[j]) , "." , as.character(scramble_name[k])	,
".MDS.pdf" ,sep=""))
	plotMDS(count_obj , col = c(rep("red",2), rep("black",6)) , top = 1000)
	invisible(dev.off())

	#------- Desigin matrix-----------------------------------------------

	ASO <- factor(c( as.character(Crtl_lib_detail$manual_sample_info.perturb_id) ,
as.character(ASO_lib_detail$manual_sample_info.perturb_id)),levels=unique(c(
as.character(Crtl_lib_detail$manual_sample_info.perturb_id) ,
as.character(ASO_lib_detail$manual_sample_info.perturb_id))))
	design <- model.matrix(~0+ASO)
	colnames(design) <- levels(ASO)

	write.table (design, file=paste ( dir_name , as.character(gene_name[j]) , "." ,
as.character(scramble_name[k])	, ".design.txt" ,sep="") , sep="\t", col.names = TRUE, quote =
FALSE, row.names = TRUE)

	#------Biological coffcient of variation (BCV) plot --------------------

	pdf( paste ( dir_name , as.character(gene_name[j]) , "." , as.character(scramble_name[k])	,
".dispersion.pdf" ,sep=""))
	count_obj <- estimateDisp(count_obj, design, robust=TRUE)
	plotBCV(count_obj)
	invisible(dev.off())

	#----------Differential expression analysis-----------------------------------------------
	fit <- glmFit(count_obj, design , robust = TRUE)
	glm_obj <- glmLRT(fit,contrast = c(-1,0.3333,0.3333,0.3333))
	DE_all <- topTags(glm_obj , n=10000000000000)
	write.table (DE_all$table, file=paste ( dir_name , as.character(gene_name[j]) , "." ,
as.character(scramble_name[k])	, ".DE_genes.txt" ,sep="") , sep="\t", col.names = TRUE, quote =
FALSE, row.names = FALSE)

	#----------CPM count-----------------------------------------------------------------------
	cpm_value_obj <- cpm(count_obj)
	cpm_value_raw <- cpm(count_data)
	write.table (cbind(count_obj$genes , cpm_value_obj), file=paste ( dir_name ,
as.character(gene_name[j]) , "." , as.character(scramble_name[k])	, ".DE_normalized_cpm.tsv"
,sep="") , sep="\t", col.names = TRUE, quote = FALSE, row.names = FALSE)
	write.table (cbind(raw_count[,1], cpm_value_raw), file=paste ( dir_name ,
as.character(gene_name[j]) , "." , as.character(scramble_name[k])	, ".DE_raw_cpm.tsv" ,sep="") ,
sep="\t", col.names = TRUE, quote = FALSE, row.names = FALSE)

	#---------Summary file-----------------------------------------------------------------------
	DGE_summary <- decideTestsDGE(glm_obj, p.value=0.05)
```

```
      c1 <- "Sample information"
      c2 <- "~~~~~~~~~~~~~~~~~~~~~~~~~~~~~~~~~~~~~~~~~~~~~~~~~~~~~~~~~~~~"
      c3 <- "DE gene summary"

      sink(file = paste ( dir_name , as.character(gene_name[j]) , "." , as.character(scramble_name[k])   ,
".DE_summary.txt" ,sep=""))
      cat (c1)
      cat("\n")
      cat (c2)
      cat("\n")
      print (count_obj$samples)
      cat("\n")
      cat (c2)
      cat("\n")
      cat (c3)
      cat("\n")
      cat (c2)
      cat("\n")
      print (summary(DGE_summary))
      cat("\n")
      cat (c2)
      sink(file=NULL)

      DE_genes <- DE_all$table
      DE_genes_FDR_flt <- DE_genes [ DE_genes$FDR <= 0.05 , ]
    }
  }
}
```
